# Semi-supervised peak calling with SPAN and JBR genome browser

**DOI:** 10.1093/bioinformatics/btab376

**Published:** 2021-05-21

**Authors:** Oleg Shpynov, Aleksei Dievskii, Roman Chernyatchik, Petr Tsurinov, Maxim N Artyomov

**Affiliations:** JetBrains Research Department, Space Office Center. Address: Primorskiy pr. 70, building 1, 197374, St.Petersburg, Russia; Department of Pathology and Immunology, 660 S Euclid Ave, St. Louis, MO 63110, USA; JetBrains Research Department, Space Office Center. Address: Primorskiy pr. 70, building 1, 197374, St.Petersburg, Russia; JetBrains Research Department, Space Office Center. Address: Primorskiy pr. 70, building 1, 197374, St.Petersburg, Russia; Department of Pathology and Immunology, 660 S Euclid Ave, St. Louis, MO 63110, USA; JetBrains Research Department, Space Office Center. Address: Primorskiy pr. 70, building 1, 197374, St.Petersburg, Russia; Department of Pathology and Immunology, 660 S Euclid Ave, St. Louis, MO 63110, USA; Department of Pathology and Immunology, 660 S Euclid Ave, St. Louis, MO 63110, USA

## Abstract

The widespread application of ChIP-seq led to a growing need for consistent analysis of multiple epigenetics profiles, for instance, in human studies where multiple replicates are a common element of design. Such multi-samples experimental designs introduced analytical and computational challenges. For example, when peak calling is done independently for each sample, small differences in signal strength/quality lead to a very different number of peaks for individual samples, making group-level analysis difficult. On the other side, when samples are pooled together for joint analysis, individual-level statistical differences are averaged out. Recently, we have demonstrated that a semi-supervised peak calling approach (SPAN) allows for robust analysis of multiple epigenetic profiles while preserving individual sample statistics. Here, we present this approach’s implementation, centered around the JBR genome browser, a stand-alone tool that allows for accessible and streamlined annotation, analysis and visualization. Specifically, JBR supports graphical interactive manual region selection and annotation, thereby addressing supervised learning’s key procedural challenge. Furthermore, JBR includes the capability for peak optimization, i.e. calibration of sample-specific peak calling parameters by leveraging manual annotation. This procedure can be applied to a broad range of ChIP-seq datasets of different quality and chromatin accessibility ATAC-seq, including single-cell experiments. JBR was designed for efficient data processing, resulting in fast viewing and analysis of multiple replicates, up to thousands of tracks. Accelerated execution and integrated semi-supervised peak calling make JBR and SPAN next-generation visualization and analysis tools for multi-sample epigenetic data.

**Availability and implementation:**

SPAN and JBR run on Linux, Mac OS and Windows, and is freely available at https://research.jetbrains.org/groups/biolabs/tools/span-peak-analyzer and https://research.jetbrains.org/groups/biolabs/tools/jbr-genome-browser.

**Supplementary information:**

Supplementary data are available at *Bioinformatics* online.

## 1 Introduction

ChIP-seq (Landt *et al.*, 2012) is the standard method to identify genome-wide DNA-binding sites for transcription factors and histone modifications. This technique’s widespread application led to a growing need to analyse experiments that contain multiple biological replicates.

Peak calling is one of the fundamental steps of ChIP-seq analysis followed by motif analysis, gene set enrichment, comparison of different conditions, etc. The most widely used tools for peak calling—MACS2 (Zhang *et al.*, 2008) and SICER (Zang *et al.*, 2009) perform peak calling independently for each replicate. This typically results in the high variability in terms of peaks called for individual samples. One example of such a situation can be seen in the recent work (Schukina *et al.*, 2020) that performed massive chromatin profiling of healthy aging in human classical monocytes comparing ∼20 young and 20 old individuals. Ultra-low input ChIP-seq approach (Brind’Amour *et al.*, 2015) allowed to overcome the constraint of having limited available material, albeit at the cost of higher levels of background noise. In this study, MACS2 peak calling pipeline produced from 5000 to 18 000 peaks for promoter-associated mark H3K4me3 in samples of same cell type taken from different individuals, and up to 3× fold change in peaks number for other histone marks, which made application of standard peak calling tools for group-level analysis impossible.

We have demonstrated that the semi-supervised peak calling approach [SPAN, (Schukina *et al.*, 2020)] can calibrate sample-specific parameters and produce a consistent number of peaks across samples with substantially different signal-to-noise ratios. In this approach, the user manually annotates a handful of genomic regions as peaks, shores and valleys. This markup is used to assess peaks significance according to the signal-to-noise ratio across samples for highly reproducible peak calling. Furthermore, the manual annotation has been shown to improve peak calling even in a single replicate setup (Hocking *et al.*, 2017; Mogilenko *et al.*, 2021).

However, the annotation-based approach is not widely used due to the annotation procedure’s technical complexity, even though conceptually it is reasonably straightforward. The most intuitive annotation would occur by marking the regions within the genome browser window, yet none of the current tools provides such capability. Here, we provide an implementation of the SPAN that is centered around JBR genome browser, which provides the user-friendly graphical ability to perform region annotation and downstream analysis/optimization using such annotation. JBR is integrated with SPAN, MACS2 and SICER peak calling methods and allows for peak optimization for either of these tools.

## 2 Materials and methods

### 2.1 General setup

**Fig. 1.**
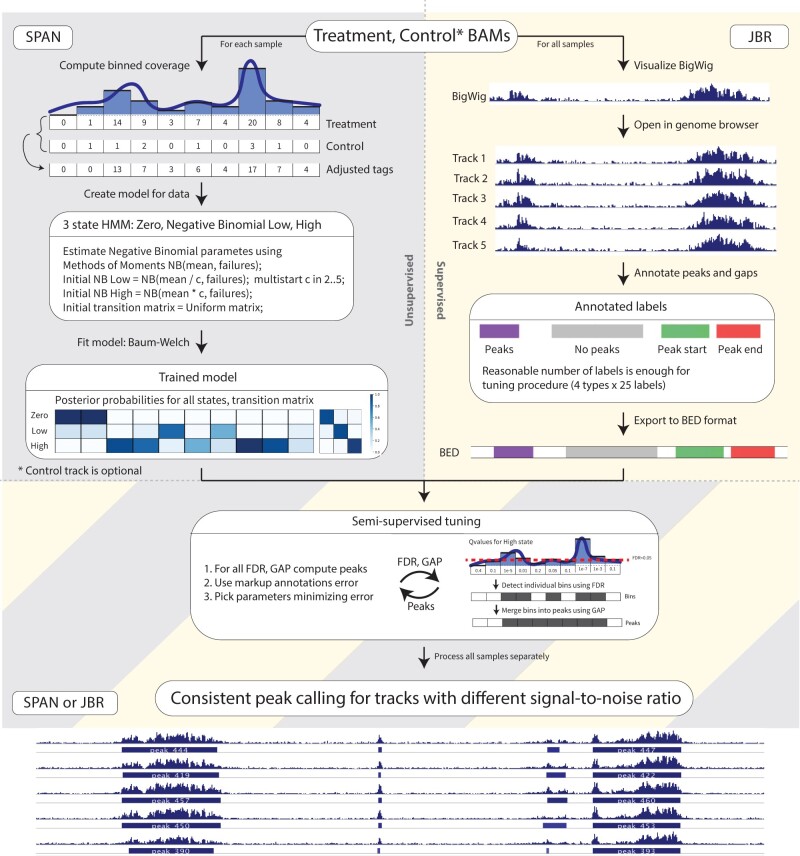
General scheme of semi-supervised peak calling pipeline with SPAN and JBR Genome Browser. 1) On the left - unsupervised SPAN model training for each individual sample. Coverage is computed for genome split into consequent non-overlapping windows. Optional control track coverage is scaled down proportionally to the treatment coverage and is subtracted from the treatment. SPAN 3 state HMM is trained with EM Baum-Welch algorithm. 2) On the rigth - supervised annotation markup creation in JBR. User uploads bigwig visualization of tracks and creates handful of annotations - peaks, no peaks, peak start, peak end. 3) SPAN model and annotation markup is used in the semi-supervised hyperparameters tuning procedure. FDR and GAP parameters are used to detect enriched windows from HMM (red dash line visualizes statistical false discovery rate of 0.05) and merge close windows into peaks. FDR and GAP combnination is optimized to minimize total number of unsatisfied markup annotations and produce final peaks track

The implemented pipeline includes the generation of the statistical model using SPAN algorithm that is then passed to for analysis to JBR genome browser (Fig. 1). The typical routine implies building a SPAN model in a Linux environment or online using Galaxy (Blankenberg *et al.*, 2010) and can be readily incorporated into a standard peak calling bioinformatics pipeline (Supplementary Fig. S1). The model output is then transferred to a stand-alone visualization tool JBR genome browser, where annotation is done directly over the visual representation of genomic coordinates and ChIP-seq signal. Given the model and annotation, peak calling can be executed on-the-fly in the JBR with immediate visualization of the peaks and signals (Supplementary Fig. S2–A).

### 2.2 Semi-supervised peak analyzer

SPAN is a semi-supervised-ready peak caller capable of processing a broad range of ChIP-seq experiments. SPAN procedure consists of two parts: (i) building a model for the signal and (ii) peak calling based on the model and manual user-supplied markup. SPAN accepts alignment files in BAM, BED or BED.GZ formats. First, the file is filtered for redundant duplicate reads to avoid possible PCR duplication artifacts. Next, SPAN estimates library fragment size by cross-correlation (Kharchenko *et al.*, 2008) and shifts sense and antisense strand tags toward the center of the fragment, which improves the spatial resolution of predicted binding sites. Afterward, it splits each chromosome into small non-overlapping bins (default length 200 bp) and operates with aggregated coverage based on the number of tags in each bin. Optional control track is used to correct possible chromatin biases in ChIP-seq experiment, including the sequence-dependent PCR amplification bias. SPAN models genome-wide chromatin coverage using Hidden–Markov Model with three states (Fig. 1). Zero state corresponds to empty bins, and two negative binomial states allow for processing experiments with arbitrary signal-to-noise ratio, gently separating signal from noise. Hidden states and parameters are inferred using the Baum–Welch algorithm (Baum *et al.*, 1970). *P*-values for bins are calculated using posterior probabilities of zero or noise states (Storey and Tibshirani, 2003). SPAN applies the Benjamini–Hochberg procedure (Benjamini and Hochberg, 1995) to estimate *q*-values and limit false discovery rate at the desired level. These *q*-values serve as model output, used for downstream semi-supervised peak calling procedure (Supplementary Fig. S2–B).

### 2.3 JBR genome browser

One of the significant challenges of supervised learning is the procedural complexity of manual data annotation, which often leads to inaccuracies and prevents widespread usage of these approaches.

JBR genome browser supports capabilities of classical genome browsers like IGV (Thorvaldsdóttir *et al.*, 2013), and provides integrated manual peak annotation with SPAN peak calling features. In the annotation editor mode, the user manually labels several genomic regions with four types of markup annotations: *peaks*, *noPeaks*, *peakStart* and *peakEnd* (Supplementary Fig. S2–C). Annotation *peaks* requires at least one peak in the marked region and guards against too conservative calling; vice versa, *noPeaks* is used against liberal peak calling; *peakStart* and *peakEnd* annotations presume single left or right peak boundary in the area, these are used to ensure appropriate peak lengths. We find that 10–20 annotations of each type are enough to produce high-quality peaks (Schukina *et al.*, 2020). After markup is ready, the user can upload SPAN model from the same interface and launch peak calling parameters optimization as a part of the JBR session. SPAN optimizes FDR threshold and gap size (distance when bins with a signal are merged) to minimize the total number of unsatisfied annotations. Moreover, JBR genome browser also supports score threshold optimization and filtering of peaks produced by MACS2 or SICER by calibrating significance level according to provided annotation markup. Of note, SPAN optimization routine for supplied markup can also be performed as stand-alone command line procedure without need for JBR, which can be useful for batch processing and computational cluster environments.

Lastly, a common challenge of processing multi-sample datasets is the need to view and analyse multiple tracks and multiple locations within a single session. Architecture based on modern Kotlin programming language and cutting-edge concurrent computing technology makes JBR smoothly load and operate with large sessions up to thousands of tracks. JBR has several additional features such as exploration of different locations simultaneously, track statistics including overlaps, lengths, etc. Furthermore, JBR can be set up as a local web server which allows for an accessible way to share the results.

## 3 Conclusion

The described pipeline is capable of processing a broad range of ChIP-seq and ATAC-seq experiments, and it was successfully applied to conventional and low input ChIP-seq, ATAC-seq and single-cell ATAC-seq datasets (Mogilenko *et al.*, 2021; Schukina *et al.*, 2020). JBR, in combination with SPAN, can serve not only as an efficient semi-supervised peak calling engine but also as a next-generation genome browser with enhanced capabilities of viewing large sessions, observing multiple locations simultaneously, etc. Both tools can be used separately, but together they comprise an ultimate solution for epigenetic data analysis.

Small test dataset together with a step-by-step tutorial can be found at: https://github.com/JetBrains-Research/span/wiki.

## Supplementary Material

btab376_Supplementary_Data
